# Supplementary material for: Circulating Tumor DNA: The Dawn of a New Era in the Optimization of Chemotherapeutic Strategies for Metastatic Colo-Rectal Cancer Focusing on RAS Mutation
Source: Cancers (Basel). 2023 Feb 25;15(5):1473. doi: 10.3390/cancers15051473 (PMC10001242; doi:10.3390/cancers15051473)
Supplement: Supplementary file 1 [file cancers-15-01473-s001.zip › cancers-2171886-supplementary.pdf]

Table S1. The comparison of tumor-informed versus tumor-agnostic approach of circulating tumor DNA analysis

Approach	Tumor tissue	Cost*	Turn-around time*	Sensitivity*	Assay
Tumor-informed approach	Necessary	High	Long	Equivalent<br>(or high)	Signatera^{TM}
Tumor-agnostic approach	Unnecessary	Low	Short	Equivalent	Caris Assure^{TM}<br>Guardant360® assay<br>FoundationOne® Liquid

*: Comparing tumor-informed approach to tumor-agnostic approach

Table S2. Previous literatures about associations between prognosis and baseline the amount of cfDNA or variant (mutant) allele frequency

References	Population	Patients number	Methodology	Prognostic factor	Outcome	Details
Tabernero J, et al [60]	mCRC	503	BEAMing	*KRAS* MAF	OS	High *KRAS* MAF was associated with shorter median OS compared to low *KRAS* MAF (cut off: median value).
Manca P, et al [61].	*RAS* WT mCRC	135	NGS	Maximum VAF	OS	Patients with high VAF had poorer median OS compared to those with low VAF (cut off: median value).
Janku F, et al [62].	Advanced cancer including mCRC	55 (mCRC; 14)	Ultra-deep NGS	Aggregate cfDNA VAF	OS	A high VAF in mutant cfDNA corresponded to shorter median OS (cut off: 6%).
El Messaoudi S, et al [63].	mCRC	97	Quantitative PCR	Mutant cfDNA concentration	OS	High mutant cfDNA concentrations were correlated with shorter median OS (cut off: median value)
Pairawan S, et al [64].	Advanced cancer including mCRC	240 (mCRC; 13)	NGS	Maximum VAF	OS	VAF was independent predictors of worse OS in multivariate analysis (cut off: the upper quartile).
Garlan F, et al [65].	mCRC	82	Droplet digital PCR	ctDNA concentration	OS	Patients with a high (>10 ng/mL) ctDNA concentration at baseline had a shorter median OS than those with a low ctDNA concentration (<0.1 ng/mL).
Peeters M, et al [66].	*RAS* WT mCRC	235	NGS	*RAS* MAF	OS	Patients with low MAF had long survival in the continuous analysis.

mCRC: metastatic colorectal cancer; WT: wild-type; NGS: next generation sequencer; PCR: polymerase chain reaction; VAF: variant allele frequency; MAF: mutant allele frequency; OS: overall survival; ctDNA: circulating tumor DNA; cfDNA: circulating cell free DNA
